# Structural mechanism of TRPC3 channel activation by the moonwalker mutation

**DOI:** 10.1038/s41421-026-00936-y

**Published:** 2026-10-29

**Authors:** Jiahe Zang, Yongmei Tan, Yikun Chen, Wenjun Guo, Xiaole Zhao, Hailin Peng, Lei Chen

**Affiliations:** 1 State Key Laboratory of Membrane Biology, College of Future Technology, Beijing Key Laboratory of Cardiometabolic Molecular Medicine, Institute of Molecular Medicine, Peking University, Beijing, China; 2 Academy for Advanced Interdisciplinary Studies, Peking University, Beijing, China; 3 Center for Nanochemistry, Beijing Science and Engineering Center for Nanocarbons, Beijing National Laboratory for Molecular Sciences, College of Chemistry and Molecular Engineering, Peking University, Beijing, China; 4 Peking-Tsinghua Center for Life Sciences, Peking University, Beijing, China; 5 National Biomedical Imaging Center, Peking University, Beijing, China

**Keywords:** Structural biology, Mechanisms of disease

Dear Editor,

TRPC3 is a receptor-activated, non-selective cation channel expressed in the cerebellum and is critical for the proper development and function of Purkinje cells^1^. A gain-of-function mutation of TRPC3, T561A, leads to impaired dendritic arborization of Purkinje cells and cerebellar ataxia, manifesting as a distinctive moonwalker gait abnormality in mice^1^. Additionally, TRPC3 has increasingly been recognized for its involvement in immune responses, cancer progression, and various cardiovascular and neurological disorders, further underscoring its potential as a therapeutic target^2^.

TRPC3 is directly activated by the second messenger diacylglycerol (DAG)^2^. Early structural studies on the TRPC3 channel revealed a tetrameric assembly consisting of a transmembrane domain (TMD) and a large cytosolic domain (CTD)^3–5^. Subsequent high-resolution structures identified key regulatory elements, including an inhibitory calcium-binding site (CBS1) within the CTD that mediates negative calcium feedback, a CBS2 with unknown function, and an activating CBS3 located in the voltage-sensor-like domain (VSLD) of the TMD^6^. Most recently, the binding modes of several TRPC3 agonists — including the endogenous DAG, the synthetic small molecule GSK1702934A, and 4n — have been characterized, providing molecular insights into how agonists interact with the channel^7^. Despite these advances, a critical gap in our understanding persists: all available structures of TRPC3, as well as its closely related homolog TRPC6 — including those bound to agonists — have captured the channel in a closed, non-conducting state. Thus, the precise structural rearrangements that drive TRPC3 channel opening remain enigmatic.

We recorded the whole-cell currents of TRPC3 elicited by the water-soluble DAG analog oleoyl-2-acetyl-sn-glycerol (OAG), showing that the current exhibited a time course characteristic of typical ligand-gated ion channels, with slow but distinct activation and desensitization phases (Fig. 1a). Although all previously determined TRPC3 structures with resolution better than 3.5 Å contain agonists at the DAG-binding L2 site, which is located at the subunit interface^3,4,6,7^, the conformations of their pores are in the same non-conductive, closed state. They may represent a pre-open state — where the agonist is bound but the channel has not yet transitioned to the open state — or a desensitized state, in which the pore closes following agonist-induced opening. To distinguish these two possibilities, we sought to determine the structure of human TRPC3 (hTRPC3) in the absence of agonist to capture its resting state. We first generated a mutant construct (hTRPC3-3M) harboring three mutations — E603A, V637A, and K607A — that are critical for DAG binding^7^. This construct could be activated by the synthetic activator GSK1702934A which also binds to the DAG-binding L2 site^7^, but showed no activation by OAG (Fig. 1b; Supplementary Fig. S1a), confirming that the mutant channel is correctly folded but its DAG-binding ability is severely impaired, and that hTRPC3-3M does not lock the channel in a desensitized state. However, we could not exclude the possibility that the synthetic agonist GSK1702934A can reactivate a desensitized hTRPC3 channel. To minimize artifacts associated with detergent solubilization, we purified the hTRPC3-3M protein using 4F-peptide solubilization followed by nanodisc assembly^8^. We determined its structure at a resolution of 2.8 Å (Supplementary Fig. S2 and Table S1). As expected, no DAG density was observed at the L2 site because of the mutations (Supplementary Fig. S2j). Instead, the L2 site was occupied by densities with different shapes (Supplementary Fig. S2j), indicating that the protein is free of agonists. Importantly, this structure is overall similar (RMSD: 0.499 Å) to the DAG-bound wild-type hTRPC3 channel purified using the same method (Supplementary Fig. S2l)^8^. We designate the hTRPC3-3M structure as the agonist-free closed state, and the previous DAG-bound structure (exemplified by PDB ID: 9VFI) as the agonist-bound closed state.

**Fig. 1:**
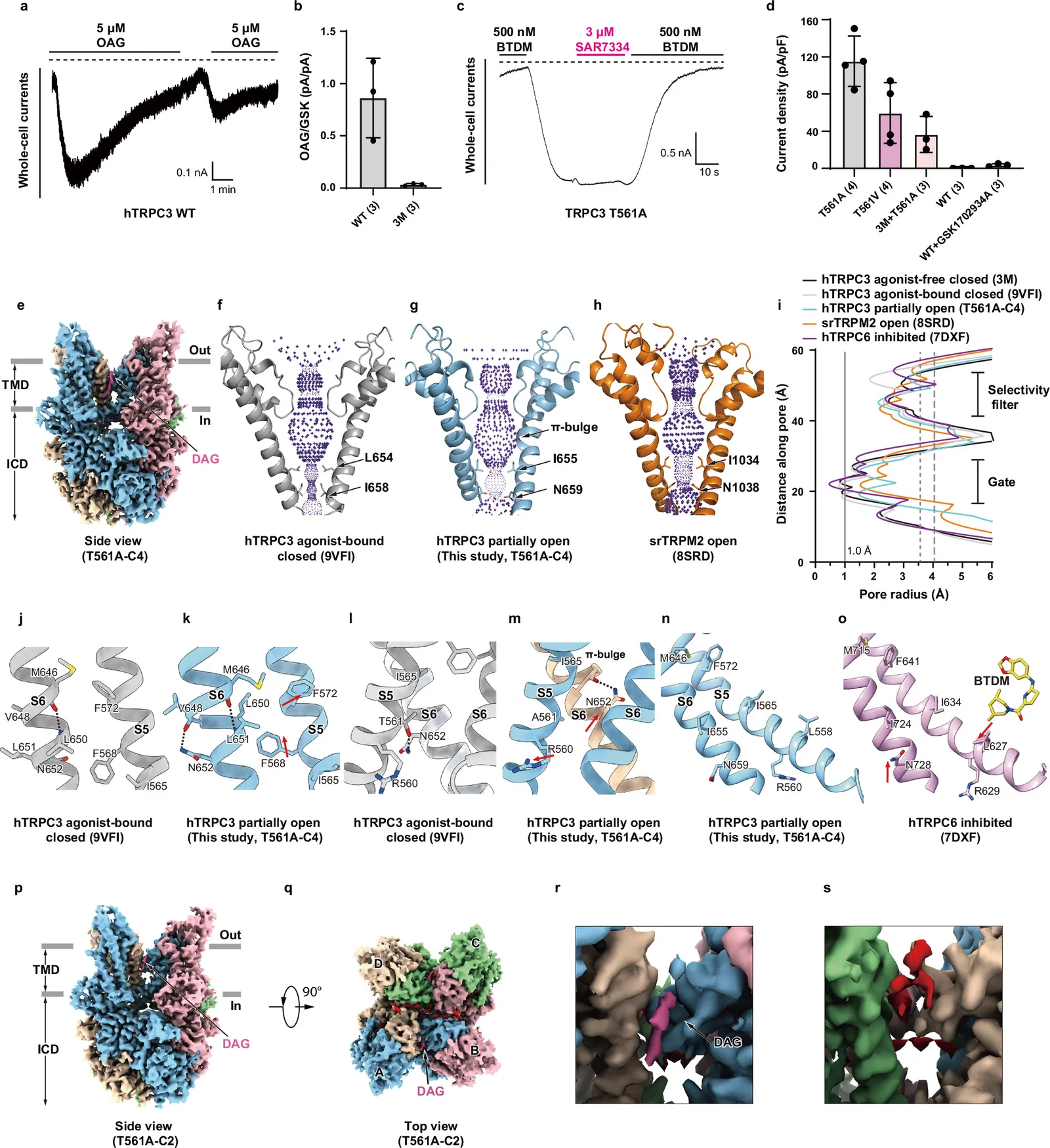
The structure of the hTRPC3 T561A mutant in the partially open state. **a** Whole-cell currents of wild-type hTRPC3 recorded at –60 mV. The dashed line denotes the zero current level. Recordings were repeated three times with similar results. **b** Ratio of whole-cell peak currents activated by 5 μM OAG and 20 μM GSK1702934A in cells transfected with wild-type hTRPC3 and hTRPC3-3M (E603A + V637A + K607A) mutant recorded at –60 mV and 48 h post-transfection. Data are presented as mean ± SD. The number of replicates in each group is indicated in parentheses. **c** Whole-cell currents of the hTRPC3 T561A mutant recorded at –20 mV. The dashed line denotes the zero current level. Recordings were repeated three times with similar results. **d** Whole-cell current densities recorded at –20 mV and 16 h post-transfection. Data are presented as mean ± SD. 20 μM GSK1702934A was used to elicit the current of wild-type hTRPC3. The numbers of biological replicates in each group are indicated in parentheses. **e** Cryo-EM density map of the hTRPC3 T561A mutant (T561A-C4, side view). Subunits A, B, C, and D are colored blue, pink, green, and yellow, respectively. DAG is colored purple. **f** Pore profile of hTRPC3 in the agonist-bound closed state (PDB ID: 9VFI) calculated using HOLE2^12^. Only the S6 helices of two diagonal subunits are shown for clarity. Residues at the pore constriction are shown as sticks. **g** Pore profile of hTRPC3 in the partially open state (T561A-C4) calculated using HOLE2. **h** Pore profile of srTRPM2 in the partially open state (PDB ID: 8SRD) calculated using HOLE2. **i** Pore radius calculated using HOLE2. The gray solid line denotes the radius of a dehydrated calcium ion (1.0 Å). The gray dashed lines denote the radii of hydrated Na^+^ (dotted) and Ca^2+^ (dashed) ions. **j** Close-up view of hTRPC3 in the agonist-bound closed state. Hydrogen bonds are denoted with dashed lines. **k** Close-up view of hTRPC3 in the partially open state. **l** Close-up view of hTRPC3 in the agonist-bound closed state. Helices from different subunits are shown in gray and silver, respectively. **m** Close-up view of hTRPC3 in the partially open state. Helices from different subunits are shown in blue and yellow, respectively. **n** Close-up view of hTRPC3 in the partially open state. **o** Close-up view of hTRPC6 in the BTDM-inhibited closed state. **p** Cryo-EM density map of the hTRPC3 T561A mutant (T561A-C2, side view). Subunits A, B, C, and D are colored blue, pink, green, and yellow, respectively. DAG is colored purple. **q** Cryo-EM density map of the hTRPC3 T561A mutant (T561A-C2, top view). Subunits A, B, C, and D are colored blue, pink, green, and yellow, respectively. Unmodeled residual densities are shown in red. DAG is colored purple. **r** Close-up view of the intact L2 DAG-binding site between subunits A–D and B–C (boxed in **q**). **s** Close-up view of the disrupted L2 DAG-binding site between subunits A–B and C–D (boxed in **q**).

We further found that although the gain-of-function T561A mutant showed a surface expression level similar to that of the wild-type hTRPC3, it exhibited constitutive currents (Fig. 1c; Supplementary Fig. S1b, c) with markedly larger current densities than wild-type hTRPC3 activated by GSK1702934A (Fig. 1d), suggesting its profoundly higher activity. Moreover, the constitutive current of T561A is sensitive to the high-affinity inhibitor BTDM^4^ but not to SAR7334^9^ (Fig. 1c; Supplementary Materials). Therefore, we sought to determine the structure of the T561A mutant because of its high activity. To minimize the cytotoxicity associated with the constitutive activity of hTRPC3 T561A during cell culture, we expressed the T561A mutant in the presence of the inhibitor BTDM, which was extensively washed away during protein purification. The hTRPC3 T561A protein was also purified using 4F-peptide solubilization followed by nanodisc assembly for cryo-EM analysis (Supplementary Figs. S3, S4, and Table S1).

Image processing yielded two major 3D classes that share an identical CTD (Supplementary Fig. S4r) but differ in their TMDs. One structure exhibits canonical C4 symmetry and was resolved at 2.93 Å resolution, representing a pore-dilated state (referred to as T561A-C4; Fig. 1e–i; Supplementary Figs. S3, S4, and Table S1), while the other exhibits C2 symmetry and was resolved at 3.16 Å resolution, representing a distorted non-conductive state (referred to as T561A-C2; Supplementary Figs. S3, S4 and Table S1). There are also several intermediate conformations that we could not resolve due to their limited resolution.

The pore profile of the agonist-bound closed state is very similar to the agonist-free closed state with a closed gate (Fig. 1i). In contrast, the T561A-C4 structure shows a dilation of the pore with an increase in the radius from 0.8 Å to 1.5 Å at the narrowest constriction (Fig. 1e–i). However, the observed pore dilation in the T561A-C4 structure is insufficient for efficient ion permeation, likely representing an intermediate conformation between the closed state and the fully open state. In this conformation, the constriction is lined by the side chains of the hydrophobic residue I655 and the hydrophilic residue N659 of hTRPC3 (Fig. 1g) — a configuration distinct from that in the agonist-bound closed state (Fig. 1f). The pore radius and pore-lining residues are similar to those observed in the open state of TRPM2 reported recently (PDB ID: 8SRD; Fig. 1h)^10^. Thus, we refer to this structure as the agonist-bound partially open state of hTRPC3. In this structure, the L2 site is occupied by DAG (Supplementary Fig. S4s). Calcium is bound in CBS3 (Supplementary Fig. S4i). A comparison of this partially open state structure with the agonist-bound closed state (PDB ID: 9VFI)^8^ revealed that their CTDs are identical (Supplementary Fig. S3m). Therefore, we focused our analysis on the TMD, where the major structural differences are observed in the S5 and S6 helices (Supplementary Video S1).

In the agonist-bound closed state, the S6 helix adopts a canonical α-helical conformation (Fig. 1f). The carbonyl group of M646 forms a hydrogen bond with the amino group of L650 (Fig. 1j), and N652 on S6 forms a hydrogen bond with T561 on S5 (Fig. 1l). In the T561A mutant, however, this N652–T561 interaction is disrupted (Fig. 1m). We further found that T561V mutant also exhibited robust constitutive currents (Fig. 1d). Valine is similar in size to threonine but cannot form a polar interaction with N652, supporting the conclusion that channel activation primarily involves the disruption of the N652–T561 interaction.

In the agonist-bound partially open state, a π-bulge is formed in the S6 helix, and the carbonyl group of M646 forms a hydrogen bond with the amino group of L651 (Fig. 1k). N652 instead forms a new polar interaction with the main-chain carbonyl group of V648, stabilizing the π-bulge (Fig. 1k). Consequently, the lower half of S6 undergoes both rotation and tilting. The rotation of S6 reconfigures not only the pore-lining residues (Fig. 1f, g) but also local interactions between S6 and its adjacent S5 helix (Fig. 1l, m), leading to concomitant conformational changes in S5. We observed the rotation of the side chains of residues R560, I565, F568, and F572 (Fig. 1m). The outward tilting of the S6 helix results in pore dilation.

BTDM is a high-affinity synthetic inhibitor of both hTRPC3 and hTRPC6^4^. We previously determined the structure of hTRPC6 in complex with BTDM at 2.9 Å resolution^6^ and found that S608L and V644F mutations in the BTDM binding pocket abolished its inhibitory effect on hTRPC6^4^. Here we found that S539L and V575F mutations in the putative BTDM-binding site of hTRPC3 also abolished the inhibitory effect of BTDM on OAG activation (Supplementary Fig. S5a), suggesting that BTDM binds hTRPC3 at the same binding site. Structural comparison between the agonist-bound partially open state structure and the hTRPC6–BTDM complex structure (Supplementary Fig. S5b–d and Video S2) suggests that upon BTDM binding, the 3,5-dimethylpiperidine group of BTDM effectively pushes the side chain and main chain of L558 on the S5 helix of hTRPC3 toward the pore center (Fig. 1n, o). In agreement with this, we found that BTDM robustly inhibits both T561A + L558A and T561A + L558F mutants in whole-cell recording (Supplementary Fig. S5e, f). Due to the intimate interactions between S5 and the adjacent gating helix S6, S6 consequently moves inward, leading to pore closure via constriction at N659 (Fig. 1n, o). Notably, in the BTDM-inhibited closed state, the π-bulge and pore-lining residues are preserved compared to the agonist-bound partially open state^6^.

In the T561A-C2 structure (Fig. 1p, q), we observed that two diagonal L2 sites (at the A–D and B–C subunit interfaces) are occupied by DAG (Fig. 1r), while the other two L2 sites (at the A–B and C–D interfaces) are disrupted and lack DAG density (Fig. 1s). As a result, the subunit interfaces associated with the unoccupied L2 sites are misaligned, leading to distortion of the selectivity filter (Supplementary Fig. S6a, b) and a rectangular gate at the bundle crossing, where L651, L654, I655, and I658 block the pore (Supplementary Fig. S6c–f). These observations suggest that DAG binding at the L2 site stabilizes the subunit interface, thereby maintaining the dilated conformation of the hTRPC3 channel. Consistent with this model, we found that reducing DAG binding by introducing hTRPC3-3M (E603A + V637A + K607A) into the hTRPC3 T561A mutant markedly decreased its constitutive currents while maintaining a similar surface expression level (Fig. 1d and Supplementary Fig. S1b, c). However, whether the C2 conformation can be converted into the C4 conformation remains elusive (Supplementary Materials).

Leveraging the structural information presented here, along with the BTDM-bound closed state structure of hTRPC6, we propose a hypothetical gating trajectory for hTRPC3 to explain the observed electrophysiological results (Supplementary Fig. S8). This trajectory encompasses transitions from the agonist-free closed state to the agonist-bound closed state, followed by agonist-stabilized pore dilation, and finally to a BTDM-inhibited closed state (Supplementary Video S3). We observed an α-to-π transition in the middle of the S6 helix, which leads to rotation and tilting of S6 and subsequent pore dilation of hTRPC3. This is similar to the opening mechanism of several other TRP channels, such as TRPV6^11^. Given the high sequence homology and likely conserved gating mechanism among TRPC3, TRPC6, and TRPC7, we speculate that this gating trajectory may be shared in this subfamily. However, we note that the agonist-free closed and agonist-bound partially open state structures were obtained using hTRPC3 mutants and may not fully recapitulate the native conformations of the wild-type channel.

## Supplementary information

Supplementary Movie 1

Supplementary Movie 2

Supplementary Movie 3

Supplemenatry information

## Data Availability

The data that support the findings of this study are available from the corresponding author upon reasonable request. Cryo-EM maps and atomic coordinates have been deposited in the EMDB and PDB databases under the accession codes EMDB: EMD-80010, EMD-80011, EMD-80012, and PDB: 25CG, 25CH, and 25CI, respectively.

## References

[1] Becker, E. B. et al. *Proc. Natl. Acad. Sci. USA* **106**, 6706–6711 (2009).

[2] Wang, H. et al. *Pharmacol. Ther.* **209**, 107497 (2020). 10.1016/j.pharmthera.2020.107497 PMC7183440 32004513

[3] Fan, C., Choi, W., Sun, W., Du, J. & Lu, W. *Elife* **7**, e36852 (2018). 10.7554/eLife.36852 PMC5967863 29726814

[4] Tang, Q. et al. *Cell Res.* **28**, 746–755 (2018). 10.1038/s41422-018-0038-2 PMC6028632 29700422

[5] Guo, W. & Chen, L. *Cell Calcium* **83**, 102075 (2019). 10.1016/j.ceca.2019.102075 31491644

[6] Guo, W. et al. *Neuron* **110**, 1023–1035.e5 (2022).

[7] Chen, Y. et al. *Nat. Commun.* **16**, 9343 (2025). 10.1038/s41467-025-64435-6 PMC12546588 41125595

[8] Zang, J. et al. *J. Mol. Biol.* **437**, 169467 (2025). 10.1016/j.jmb.2025.169467 41061950

[9] Maier, T. et al. *Br. J. Pharmacol.* **172**, 3650–3660 (2015). 10.1111/bph.13151 PMC4507166 25847402

[10] Huang, Y., Kumar, S., Lee, J., Lu, W. & Du, J. *Nat. Struct. Mol. Biol.* **31**, 1509–1521 (2024). 10.1038/s41594-024-01316-4 PMC11479946 38773335

[11] McGoldrick, L. L. et al. *Nature* **553**, 233–237 (2018). 10.1038/nature25182 PMC5854407 29258289

[12] Smart, O. S., Neduvelil, J. G., Wang, X., Wallace, B. A. & Sansom, M. S. *J. Mol. Graph.* **14**, 354–360 (1996). 10.1016/s0263-7855(97)00009-x 9195488

