# Supplementary material for: Structural mechanism of TRPC3 channel activation by the moonwalker mutation
Source: Cell Discov. 2026 Oct 29;12:75. doi: 10.1038/s41421-026-00936-y (PMC13720187; doi:10.1038/s41421-026-00936-y)
Supplement: Supplementary file 1 [file 41421_2026_936_MOESM4_ESM.pdf]

Supplementary Materials for

Structural Mechanism of TRPC3 Channel Activation by the Moonwalker Mutation

Jiahe Zang [1,6], Yongmei Tan [2,6], Yikun Chen[2], Wenjun Guo[1],

Xiaole Zhao [3], Hailin Peng [3], and Lei Chen [1,2,4,5,*]

[1] State Key Laboratory of Membrane Biology, College of Future Technology, Institute of Molecular Medicine, Peking University, Beijing Key Laboratory of Cardiometabolic Molecular Medicine, Beijing 100871, China.

[2] Academy for Advanced Interdisciplinary Studies, Peking University, Beijing 100871, China

[3] Center for Nanochemistry, Beijing Science and Engineering Center for Nanocarbons, Beijing National Laboratory for Molecular Sciences, College of Chemistry and Molecular Engineering, Peking University, Beijing 100871, China.

[4] Peking-Tsinghua Center for Life Sciences, Peking University, Beijing 100871, China

[5] National Biomedical Imaging Center, Peking University, Beijing, 100871, China

[6] These authors contribute equally to this work

*Correspondence: Lei Chen, chenlei2016@pku.edu.cn

This file includes:

Supplementary Discussion

Materials and Methods

Additional references

Supplementary Figs. 1-8

Supplementary Table 1

Supplementary Discussion

Other TRP channel structures showing C2 symmetry

It is worth noting that the C2-symmetric state was previously observed for several other homo-tetrameric TRP channel members, including TRPV channels [1-3] and TRPM channels[4-6]. In addition, the TRPV3 G568D mutant was reported to show a C2 conformation with a distorted selectivity filter as described here[7].

Differential inhibitory effects of BTDM and SAR7334 on T561A mutant

We also uncovered differential effects of hTRPC3 inhibitors on the T561A mutant. BTDM binds at the Inhibitor Binding Pocket-C (IBP-C) site [8,9], which is located between the pore and the voltage sensor-like domain (VSLD), and effectively inhibits the constitutive current of the T561A mutant (Fig. 1c). In contrast, SAR7334— which binds at the Inhibitor Binding Pocket-A (IBP-A) site in the VSLD [9]—does not inhibit the currents of the T561A mutant (Fig. 1c). This correlates with the fact that the VSLD of hTRPC3 does not show large conformational differences between the agonist-bound closed state and the agonist-bound partially open state (Supplementary Fig. 7e) and suggests that the VSLD regulates hTRPC3 activity upstream of the T561 site.

The role of DAG in the activation of TRPC3

By comparing the structures of the agonist-free closed state, the agonist-bound closed state, and the agonist-bound partially open state, we did not observe large-scale conformational changes in the binding site of the DAG glycerol head group (Supplementary Fig. 7a-c). However, the binding site for the two acyl tails of DAG is in close proximity to residues that undergo substantial rearrangements during channel opening, including F572, M569, and I565 on S5, and V649 and L650 on S6 (Supplementary Fig. 7d). Furthermore, DAG binding appears to stabilize the partially open state: the T561A-C2 structure shows that the loss of DAG at

subunit interfaces results in a distorted, non-conductive pore (Fig. 1q), and abolishing the DAG-binding site via the 3M mutations dramatically reduced currents (Fig. 1d). Together, these observations support the role of DAG as an agonist that stabilizes the open conformation of hTRPC3. It has been reported that PIP_2 binds to the L3 site to support the opening of hTRPC3 [10]. However, we did not observe clear PIP_2 density in our map, suggesting that PIP_2 was not bound in our structures, which might explain why the T561A-C4 structure is not fully open.

Comparison with others' work

During the preparation of this manuscript, a related study was published[11], which reported the closed-state structure of the T561A mutant (residue 573A in their construct; PDB ID: 9OLL, EMD-70596) and the GSK417-inhibited T561A mutant featuring an α-helical S6 segment (PDB ID: 9OLX, EMD-70601). However, careful examination of the deposited PDB models and cryo-EM density maps revealed errors in the model building of both structures. Their proposed closed-state structure of the T561A mutant (9OLL) actually corresponds to a partially open state, and the GSK417-bound T561A mutant (9OLX) contains a π-bulge within the S6 helix. Accordingly, their structural data are largely consistent with our present work.

Materials and Methods

Cell Culture

Sf9 insect cells (Thermo Fisher Scientific) were cultured in SIM SF medium (Sino Biological) at 27 °C. Adapted Expi293F cells (donated by Shanghai OPM Biosciences) were cultured in 293F Hi-exp Medium (Shanghai OPM Biosciences) at 37 °C with 6% CO_2 and 70% humidity. FreeStyle 293F suspension cells (Thermo Fisher Scientific) were cultured in FreeStyle 293 medium (Thermo Fisher Scientific) supplemented with 1% fetal bovine serum (FBS, VisTech), 67 μg/mL penicillin (Macklin), and 139 μg/mL streptomycin (Macklin) at 37 °C with 6% CO_2

and 70% humidity. All cell lines were routinely tested for mycoplasma contamination and were confirmed negative, but they have not been authenticated.

Constructs

For surface expression and electrophysiology, we generated an N-terminal GFP-tagged hTRPC3 vector without strep tag. For expression and purification of the hTRPC3-3M mutant, we generated N-terminal GFP-MBP (Maltose Binding Protein)-HRV 3C site-hTRPC3 with strep tag in a loop of GFP [12]. For expression and purification of hTRPC3-T561A mutant, we generated N-terminal GFP-MBP (Maltose Binding Protein)-HRV 3C site-hTRPC3 with strep tag after HRV 3C site [12].

Estimation of the surface expression of hTRPC3 protein

Adapted Expi293F cells were seeded into 6-well plates at a density of 2×10^6 cells/mL in 3 mL per well and transfected with wild-type hTRPC3 or its mutants using PEI. Cells transfected with an N-terminal GFP-tagged vector served as a negative control. After transfection, 400 nM BTDM was added to the medium and incubated at 37 °C for 48 h.

Cells were then collected by centrifugation at 4000 rpm for 5 min at 4 °C and washed three times with 1 mL of cold PBS (pH 8.0). After the final wash, cells were resuspended in 100 μL of cold PBS (pH 8.0), followed by the addition of 20 μL freshly prepared 10 mM Sulfo-NHS-SS-biotin (TargetMol, T19061) in PBS (pH 8.0). Cells were incubated at room temperature for 1 h. The reaction was terminated by centrifugation at 4000 rpm for 5 min at 4 °C, and cells were resuspended in 100 μL of ice-cold TBS containing 100 mM glycine (pH 8.0), followed by another round of centrifugation.

Cells were washed three times with 100 μL of cold TBS (pH 8.0), followed by centrifugation at 4000 rpm for 5 min each time. Cell pellets were then lysed on ice in 100 μL TBS containing 1% LMNG + 0.1% CHS for 40 min. Lysates were cleared by centrifugation at 16,125 × g for

20 min at 4 °C, and the supernatants were incubated with 20 μL Streptavidin agarose beads (Smart-Lifesciences, SA053100) for 2 h at 4 °C.

Beads were collected by centrifugation at 1000 × g for 2 min, and flow-through fractions were discarded. The beads were then washed four times with 50 μL of wash buffer (50 mM Tris, 150 mM NaCl, pH 8.0), with centrifugation at 1000 × g for 2 min after each wash. Proteins were eluted with 50 μL of elution buffer by breaking the disulfide bond of Sulfo-NHS-SS-biotin (50 mM Tris, pH 8.0, 150 mM NaCl, 100 mM DTT, 1% SDS) at room temperature for 2 h, followed by centrifugation at 1000 × g for 2 min.

Protein concentrations of the eluates were determined using the Bradford assay. Equal amounts of protein from each sample were separated by SDS-PAGE. Expression of N-cadherin was assessed by Western blot using antibodies against N-cadherin (1:3000) (Proteintech, 22018-1-AP). Surface expression levels of hTRPC3 and its mutants were detected by in-gel GFP fluorescence.

Electrophysiology

For whole-cell recordings of TRPC3, the pipette solution contained 120 mM CsMe (cesium methanesulfonate), 25 mM CsCl, 2 mM $MgCl_2$, 2 mM EGTA, and 30 mM HEPES (pH = 7.4, adjusted with CsOH). The bath solution contained 140 mM NaCl, 5 mM CsCl, 2 mM $MgCl_2$, 1 mM EDTA, 2 mM EGTA, 10 mM glucose, and 10 mM HEPES (pH = 7.4, adjusted with NaOH). The concentrations of 1-Oleoyl-2-acetyl-sn-glycerol (OAG, Sigma, O6754) and BTDM (donated by Dizal Pharmaceutical) used in patch-clamp experiments were indicated in the figures.

Patch electrodes were pulled using a horizontal microelectrode puller (P-1000, Sutter Instrument Co., USA) to a tip resistance of 2.0–4.0 MΩ. An MPS-2 perfusion system (Yibo Company, Wuhan, China) was used for buffer exchange. TRPC currents were recorded at a

holding potential of -20 mV or −60 mV using an Axopatch 200B amplifier (Axon Instruments, USA). Currents with amplitudes greater than 150 pA were used for further analysis. Data were low-pass filtered using pCLAMP 10.4 software. Current traces were plotted using Origin 8, and peak currents were statistically analyzed using GraphPad Prism 8.

Protein expression and purification

For expression of the hTRPC3 T561A mutant, adapted Expi293F cells were cultured in 293F Hi-exp Medium (Shanghai OPM Biosciences) at a density of 3.0 × 10^6 cells mL^{-1} and transfected with the hTRPC3 T561A mutant plasmid using polyethyleneimine (PEI MAX, Polysciences). Twelve hours post-transfection, 10 mM sodium butyrate and 400 nM BTDM were added to the medium, and cells were cultured for an additional 48 hours before harvesting. For harvesting, 4 mM EDTA was added to block divalent cations in the culture medium, and cells were washed four times with TBS buffer (20 mM Tris pH 8.0 at 4 °C, 150 mM NaCl) containing 1 mM EDTA at room temperature to remove BTDM before being frozen at −80 °C.

For purification of the hTRPC3 T561A mutant, cell pellets corresponding to 800 mL of culture were solubilized in 50 mL of lysis buffer [2 mg/mL 4F (DWFKAFYDKVAEKFKEAF), 25 mM Tris pH 8.0 at 4 °C, 5 mM DTT, 1 mM EDTA, and protease inhibitors] and rotated at 4 °C for 1 hour. After centrifugation at 40,000 rpm for 40 minutes using a Type 50.2 rotor (Beckman), the supernatant was loaded onto a column packed with amylose resin (NEB). The resin was washed with TBS buffer containing 5 mM DTT and 1 mM EDTA. Protein was eluted with MBP elution buffer (80 mM maltose, 100 mM NaCl, 20 mM Tris pH 8.0 at 4 °C, 5 mM DTT, and 1 mM EDTA) and incubated with HRV 3C protease at 4 °C overnight. The next day, the protein was incubated with MSP2N2 at a 1:10 molar ratio for 1 hour at room temperature, and the mixture was loaded onto Streptactin resin and washed with TBS containing 5 mM DTT. The target protein was eluted with Strep elution buffer (50 mM Tris pH 8.0 at 4 °C, 100 mM

NaCl, 5 mM desthiobiotin, and 5 mM DTT) and further purified using a Superose-6 Increase column (GE Healthcare) equilibrated with S6 buffer (20 mM HEPES pH 7.5, 100 mM NaCl, and 1 mM TCEP). Peak fractions containing the tetrameric hTRPC3 channel in nanodiscs were combined and concentrated for cryo-EM studies.

For expression of the hTRPC3 3M mutant, FreeStyle 293F suspension cells grown in FreeStyle 293 medium at 37 °C with a density of 2.3×10^6 cells mL^{-1} were infected with BacMam viruses. Eight to twelve hours post-infection, 10 mM sodium butyrate was added, and the temperature was lowered to 30 °C. Cells were collected 48 hours post-infection, washed twice with TBS buffer, and frozen at −80 °C.

For purification of the hTRPC3 3M mutant, cell pellets corresponding to 800 mL of culture were solubilized in 50 mL of lysis buffer (2 mg/mL 4F, 25 mM Tris pH 8.0 at 4 °C, 5 mM DTT, 1 mM EDTA, and protease inhibitors) and rotated at 4 °C for 1 hour. After centrifugation at 40,000 rpm for 40 minutes using a Type 50.2 rotor (Beckman), the supernatant was loaded onto a column packed with amylose resin (NEB). The resin was washed with TBS buffer containing 5 mM DTT and 1 mM EDTA. Protein was eluted with MBP elution buffer (80 mM maltose, 100 mM NaCl, 20 mM Tris pH 8.0 at 4 °C, 5 mM DTT, and 1 mM EDTA) and incubated with MSP2N2 at a 1:10 molar ratio at 4 °C overnight. The mixture was then loaded onto Streptactin resin and washed with TBS containing 5 mM DTT. The target protein was eluted with Strep elution buffer (50 mM Tris pH 8.0 at 4 °C, 100 mM NaCl, 5 mM desthiobiotin, and 5 mM DTT) and incubated with GST-tagged HRV 3C protease at 4 °C overnight. The next day, the protein was loaded onto a GST column. The wash and flow-through (FT) fractions were collected using TBS (pH 7.5 at room temperature) supplemented with 5 mM DTT to remove HRV 3C protease. The mixture was then further purified using a Superose-6 Increase column (GE Healthcare) equilibrated with S6 buffer (20 mM HEPES pH 7.5, 100 mM NaCl,

and 1 mM TCEP). Peak fractions containing the tetrameric hTRPC3 channel in nanodiscs were combined and concentrated for cryo-EM studies.

Cryo-EM sample preparation

For the purified hTRPC3 T561A mutant ($A_{280} \approx 0.12$), 1 mM $CaCl_2$ was added. After centrifugation at 40,000 rpm for 30 minutes, the supernatant was collected and supplemented with 0.5 mM fluorinated octyl maltoside (FOM). The protein was applied to Quantifoil (1.2/1.3) gold grids coated with graphene [13] for cryo-EM sample preparation.

For the purified hTRPC3 3M mutant ($A_{280} \approx 1.6$), 1 mM $CaCl_2$ was added. After centrifugation at 40,000 rpm for 30 minutes, the supernatant was collected and supplemented with 0.5 mM fluorinated octyl maltoside (FOM). The protein was applied to GIG (1.2/1.3) Cu grids coated with graphene [13] for cryo-EM sample preparation.

Cryo-EM data collection

Cryo-EM grids were screened using a Talos Arctica electron microscope (Thermo Fisher Scientific) operating at 200 kV with a K2 camera (Thermo Fisher Scientific). Data were collected using a 300 kV Titan Krios (Thermo Fisher Scientific) equipped with a K3 camera (Gatan) and an energy filter set to a slit width of 20 eV.

Data of the hTRPC3 T561A mutant were collected at a magnification of 47,000× (pixel size = 1.067 Å), with a defocus range of −1.5 to −1.8 μm, a total dose of approximately 50 $e^-/Å^2$, and a dose rate of 22.36 e^-/pixel/s on the detector. Data of the hTRPC3 3M mutant were collected at a magnification of 60,000× (pixel size = 0.833 Å), with a defocus range of −1.5 to −1.8 μm, a total dose of approximately 50 $e^-/Å^2$, and a dose rate of 22.15 e^-/pixel/s on the detector.

Cryo-EM data processing

The data for the hTRPC3 T561A mutant were subjected to gain correction, motion correction, anisotropic magnification correction, and dose weighting using MotionCor2-1.3.2 [14]. Selected micrographs were imported into cryoSPARC-4.5.3 [15], where a round of template picking (Template: EMD-30903), 2D classification, ab-initio reconstruction using C1 symmetry and 3D classification using C1 symmetry, was performed. We found that the majority of the particles with high resolution features showed C2 or C4 symmetry. We also found a low-resolution reconstruction with C1 symmetry. Subsequently, we used Topaz [16] for particle picking and seed-facilitated 3D classification [17] to remove "bad" particles, obtaining 225,181 "good" particles with C4 symmetry. Additional seed-facilitated 3D classification and 3D classification yielded particles with C1 symmetry (28,910 particles) and C2 symmetry (106,729 particles). Particles with C1, C2, or C4 symmetry were subjected to non-uniform refinement [18]. Resolution estimation was based on the gold standard Fourier Shell Correlation (FSC) 0.143 cutoff. Although the resolution of C1 map reached 3.78 Å, the local map quality of the TMD is insufficient for us to determine the DAG-binding state, and this map was not discussed in this work.

Data processing for the hTRPC3 3M mutant followed a similar strategy. Selected micrographs were imported into cryoSPARC-4.5.3, where a round of template picking, 2D and 3D classification, and 3D refinement was performed. Only map showing C4 symmetry was observed. Particles from the best class were used as "seeds" for seed-facilitated 3D classification [17]. Particles selected after multi-reference 3D classification were subjected to final non-uniform refinement with C4 symmetry. Resolution estimation was based on the gold standard FSC 0.143 cutoff.

Model building, refinement, and validation

hTRPC3 models were manually rebuilt using Coot-0.9.8.93 [19] based on the published model (PDB ID: 7DXC) and further refined using PHENIX 1.19.2 [20,21].

Quantification and statistical analysis

Data processing and statistical analysis were performed using GraphPad Prism software. Statistical details are provided in the methods and figure legends. Global resolution estimations of cryo-EM density maps were based on the 0.143 FSC criterion [22]. Local resolution was estimated using cryoSPARC. The number of independent experiments (N) and relevant statistical parameters (e.g., mean or standard deviation) are described in the figure legends. No statistical methods were used to pre-determine sample sizes.

Supplementary Video 1. Conformational changes of hTRPC3 from the agonist-bound closed state to the agonist-bound partially open state.

hTRPC3 is colored the same as shown in Fig. 1e. Morphs of hTRPC3 between the agonist-bound closed state (PDB ID: 9VFI) and the agonist-bound partially open state (T561A-C4) are generated using Chimera X.

Supplementary Video 2. Conformational changes of hTRPC3 from the agonist-bound partially open state to the BTDM-inhibited state.

hTRPC3 is colored the same as shown in Fig. 1e. The structure of hTRPC3 in the BTDM-inhibited state was generated with SWISS-MODEL using the structure of hTRPC6 in complex with BTDM (PDB ID: 7DXF) as the template. BTDM is shown as spheres. Morphs of hTRPC3 between the agonist-bound partially open state (T561A-C4) and the BTDM-inhibited state are generated using Chimera X.

Supplementary Video 3. A hypothetical gating trajectory of hTRPC3 during the *in vitro* whole-cell recordings.

The morphs of hTRPC3 from the agonist-free closed state (hTRPC3-3M) to the agonist-bound closed state (PDB ID: 9VFI), followed by the agonist-bound partially open state (T561A-C4), and finally to a BTDM-inhibited state (modelled from PDB ID: 7DXF) during the *in vitro* whole cell recordings.

Additional References

1 Zhang, K., Julius, D. & Cheng, Y. Structural snapshots of TRPV1 reveal mechanism of polymodal functionality. *Cell* **184**, 5138-5150 e5112 (2021). https://doi.org:10.1016/j.cell.2021.08.012

2 Zubcevic, L., Hsu, A. L., Borgnia, M. J. & Lee, S. Y. Symmetry transitions during gating of the TRPV2 ion channel in lipid membranes. *Elife* **8** (2019). https://doi.org:10.7554/eLife.45779

3 Zubcevic, L. *et al.* Conformational ensemble of the human TRPV3 ion channel. *Nat Commun* **9**, 4773 (2018). https://doi.org:10.1038/s41467-018-07117-w

4 Yin, Y. *et al.* Activation mechanism of the mouse cold-sensing TRPM8 channel by cooling agonist and PIP(2). *Science* **378**, eadd1268 (2022). https://doi.org:10.1126/science.add1268

5 Yin, Y. *et al.* Visualizing structural transitions of ligand-dependent gating of the TRPM2 channel. *Nat Commun* **10**, 3740 (2019). https://doi.org:10.1038/s41467-019-11733-5

6 Kim, S. H. *et al.* Symmetry-driven gating of TRPM8 by PIP(2) and menthol. *Nat Commun* **17** (2026). https://doi.org:10.1038/s41467-026-75793-0

7 Khau, J., Purohit, R., Nadezhdin, K. D., Talyzina, I. A. & Sobolevsky, A. I. Structural diversity of heat-sensing channel TRPV3 with Olmsted syndrome mutations. *Nat Commun* (2026). https://doi.org:10.1038/s41467-026-74687-5

8 Tang, Q. *et al.* Structure of the receptor-activated human TRPC6 and TRPC3 ion channels. *Cell Res.* **28**, 746-755 (2018). https://doi.org:10.1038/s41422-018-0038-2

9 Guo, W., Tang, Q., Wei, M., Kang, Y., Wu, J. X. & Chen, L. Structural mechanism of human TRPC3 and TRPC6 channel regulation by their intracellular calcium-binding sites. *Neuron* **110**, 1023-1035 e1025 (2022). https://doi.org:10.1016/j.neuron.2021.12.023

10 Clarke, A. *et al.* PIP(2) modulates TRPC3 activity via TRP helix and S4-S5 linker. *Nat Commun* **15**, 5220 (2024). https://doi.org:10.1038/s41467-024-49396-6

11 Bell, B. *et al.* Functional and structural basis of a hypermorphic TRPC3 variant. *Sci Adv* **12**, eaec9284 (2026). https://doi.org:10.1126/sciadv.aec9284

12 Guo, W., Wang, M. & Chen, L. A co-expression vector for baculovirus-mediated protein expression in mammalian cells. *Biochem. Biophys. Res. Commun.* **594**, 69-73 (2022). https://doi.org:10.1016/j.bbrc.2022.01.056

13 Zhang, J. *et al.* Clean Transfer of Large Graphene Single Crystals for High-Intactness Suspended Membranes and Liquid Cells. *Adv Mater* **29** (2017). https://doi.org:10.1002/adma.201700639

14 Zheng, S. Q., Palovcak, E., Armache, J. P., Verba, K. A., Cheng, Y. & Agard, D. A. MotionCor2: anisotropic correction of beam-induced motion for improved cryo-

electron microscopy. *Nat. Methods* **14**, 331-332 (2017). https://doi.org:10.1038/nmeth.4193
15 Punjani, A., Rubinstein, J. L., Fleet, D. J. & Brubaker, M. A. cryoSPARC: algorithms for rapid unsupervised cryo-EM structure determination. *Nat. Methods* **14**, 290-296 (2017). https://doi.org:10.1038/nmeth.4169
16 Bepler, T. *et al.* Positive-unlabeled convolutional neural networks for particle picking in cryo-electron micrographs. *Nat. Methods* **16**, 1153-1160 (2019). https://doi.org:10.1038/s41592-019-0575-8
17 Wang, N. *et al.* Structural basis of human monocarboxylate transporter 1 inhibition by anti-cancer drug candidates. *Cell* **184**, 370-383 e313 (2021). https://doi.org:10.1016/j.cell.2020.11.043
18 Punjani, A., Zhang, H. & Fleet, D. J. Non-uniform refinement: adaptive regularization improves single-particle cryo-EM reconstruction. *Nat. Methods* **17**, 1214-1221 (2020). https://doi.org:10.1038/s41592-020-00990-8
19 Emsley, P., Lohkamp, B., Scott, W. G. & Cowtan, K. Features and development of Coot. *Acta Crystallogr. D Biol. Crystallogr.* **66**, 486-501 (2010). https://doi.org:10.1107/S0907444910007493
20 Adams, P. D. *et al.* PHENIX: a comprehensive Python-based system for macromolecular structure solution. *Acta Crystallogr. D Biol. Crystallogr.* **66**, 213-221 (2010). https://doi.org:10.1107/S0907444909052925
21 Afonine, P. V. *et al.* Real-space refinement in PHENIX for cryo-EM and crystallography. *Acta Crystallogr D Struct Biol* **74**, 531-544 (2018). https://doi.org:10.1107/S2059798318006551
22 Chen, S. *et al.* High-resolution noise substitution to measure overfitting and validate resolution in 3D structure determination by single particle electron cryomicroscopy. *Ultramicroscopy* **135**, 24-35 (2013). https://doi.org:10.1016/j.ultramic.2013.06.004

Supplementary Figure 1

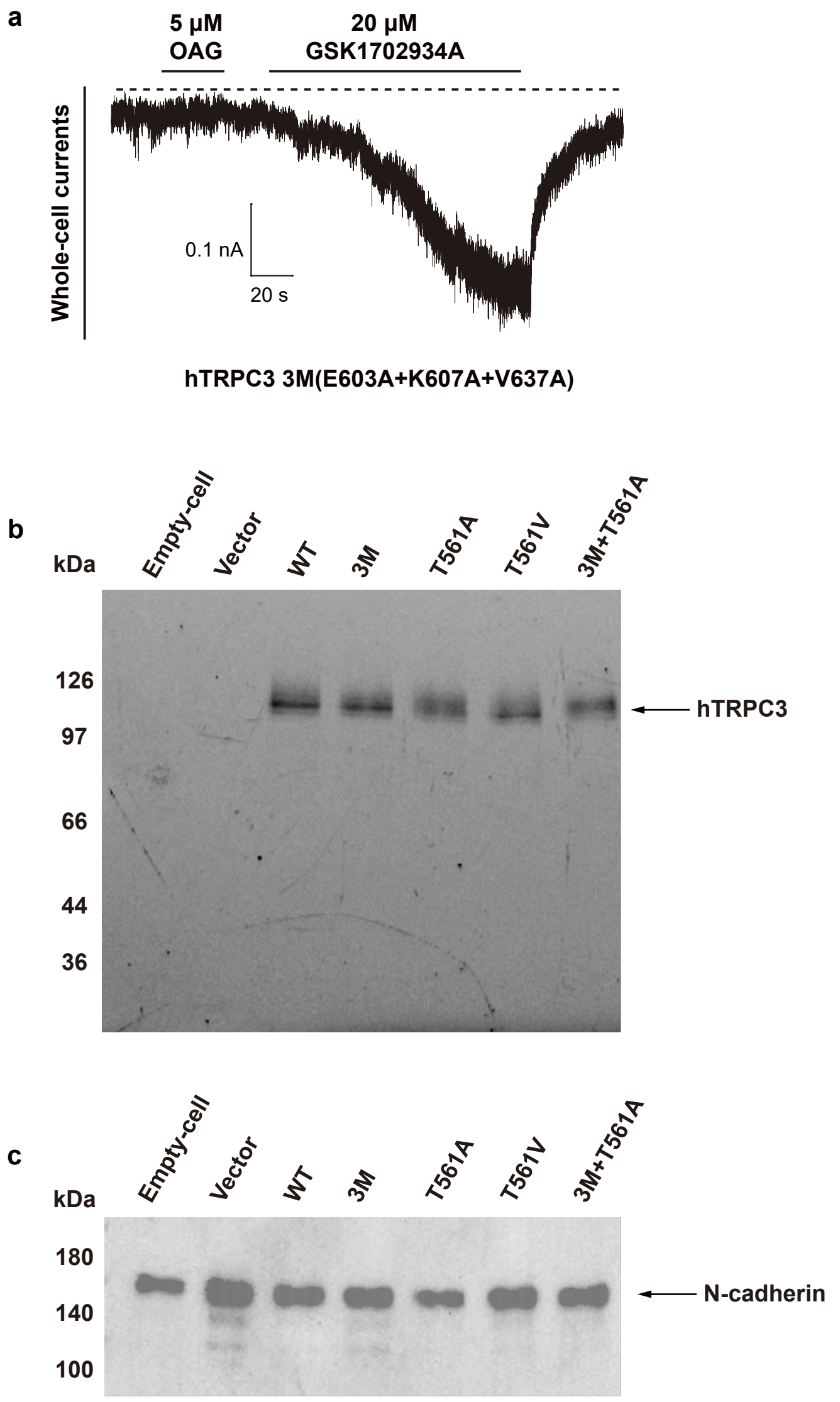

Supplementary Figure 1. Whole-cell currents of hTRPC3 3M mutant and surface expression levels of wild-type TRPC3 and its mutants.

a, Whole-cell currents of hTRPC3 3M mutant recorded at -60 mV and 48 hours post-transfection. The dashed line denotes the zero current level. Recordings were repeated three times with similar results.

b, Surface expression levels of hTRPC3 wild-type and hTRPC3 mutants determined by in-gel GFP fluorescence.

c, The surface expression levels of endogenous N-Cadherin.

Supplementary Figure 2

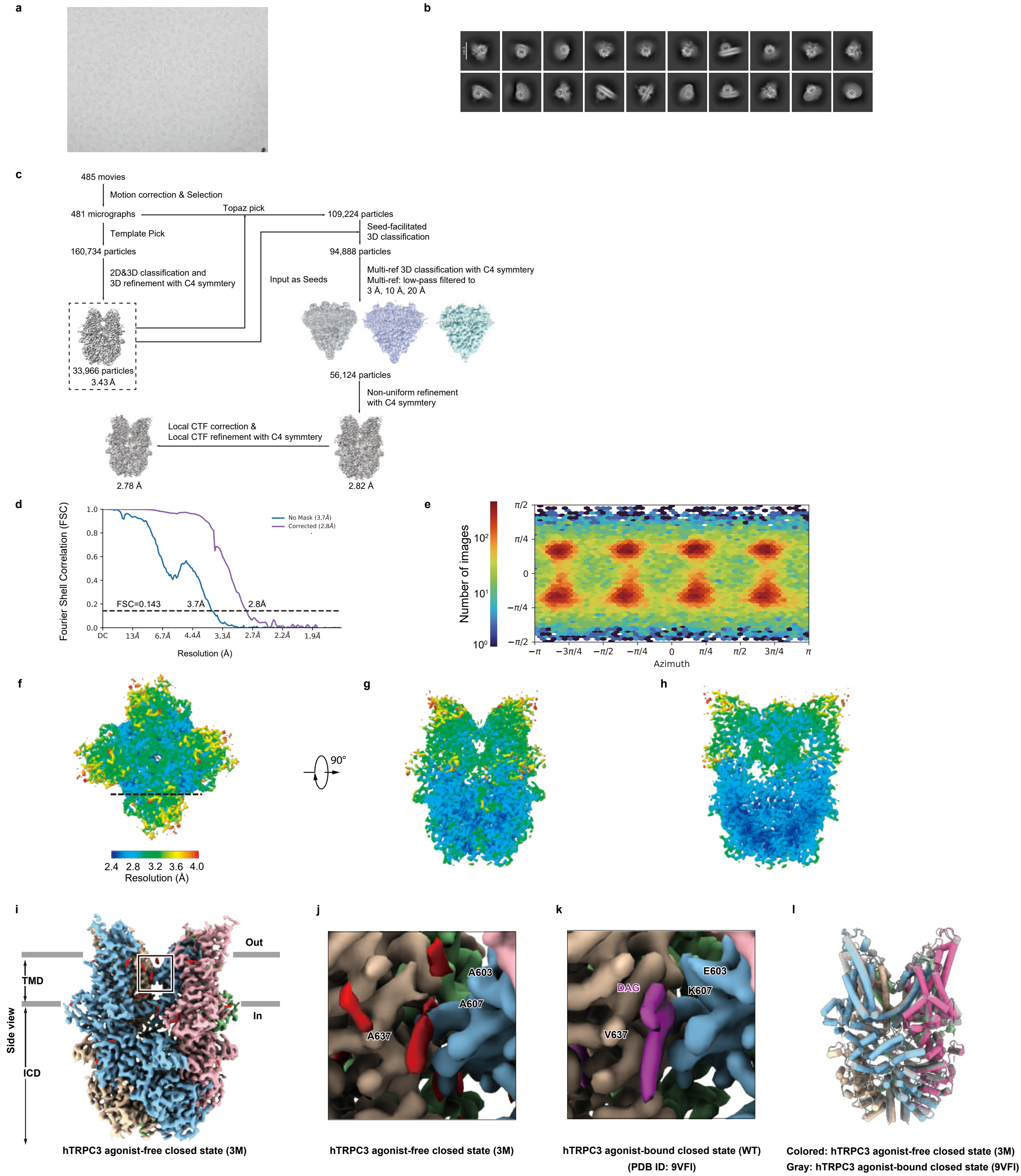

Supplementary Figure 2, Cryo-EM image analysis of hTRPC3 3M.

a, Representative raw micrograph of hTRPC3 3M purified using 4F-disc.
b, Representative 2D class average of hTRPC3 3M.
c, Flowchart of hTRPC3 3M cryo-EM data processing.
d, FSC curves of the final refinement for hTRPC3 3M after correction for masking effects. Resolution estimations were based on the FSC 0.143 cutoff criterion.
e, Angular distribution of the final refinement for hTRPC3 3M (standard output from cryoSPARC).
f, Local resolution estimation of hTRPC3 3M (top view).
g, Local resolution estimation of hTRPC3 3M (side view).
h, Cross-section of the local resolution estimation of hTRPC3 3M. The position of the cross-section is indicated by the dashed line in (f).
i, Cryo-EM density map of the hTRPC3 3M mutant (side view), contoured at 4.72 σ. Subunits A, B, C, and D are colored blue, pink, green, and yellow, respectively. Unmodeled residual densities are shown in red. The box denotes the L2 site, where the E603A, K607A, and V637A mutations are located.
j, Close-up view of the electron density at the L2 DAG-binding site of the hTRPC3 3M mutant (boxed in panel i), contoured at 4.72 σ.
k, Close-up view of the electron density at the L2 DAG-binding site of wild-type hTRPC3 (EMD-65025), contoured at 8.66 σ. DAG is colored purple.
l, Structural comparison between hTRPC3 3M and hTRPC3 WT (gray, RMSD: 0.499 Å).

Supplementary Figure 3

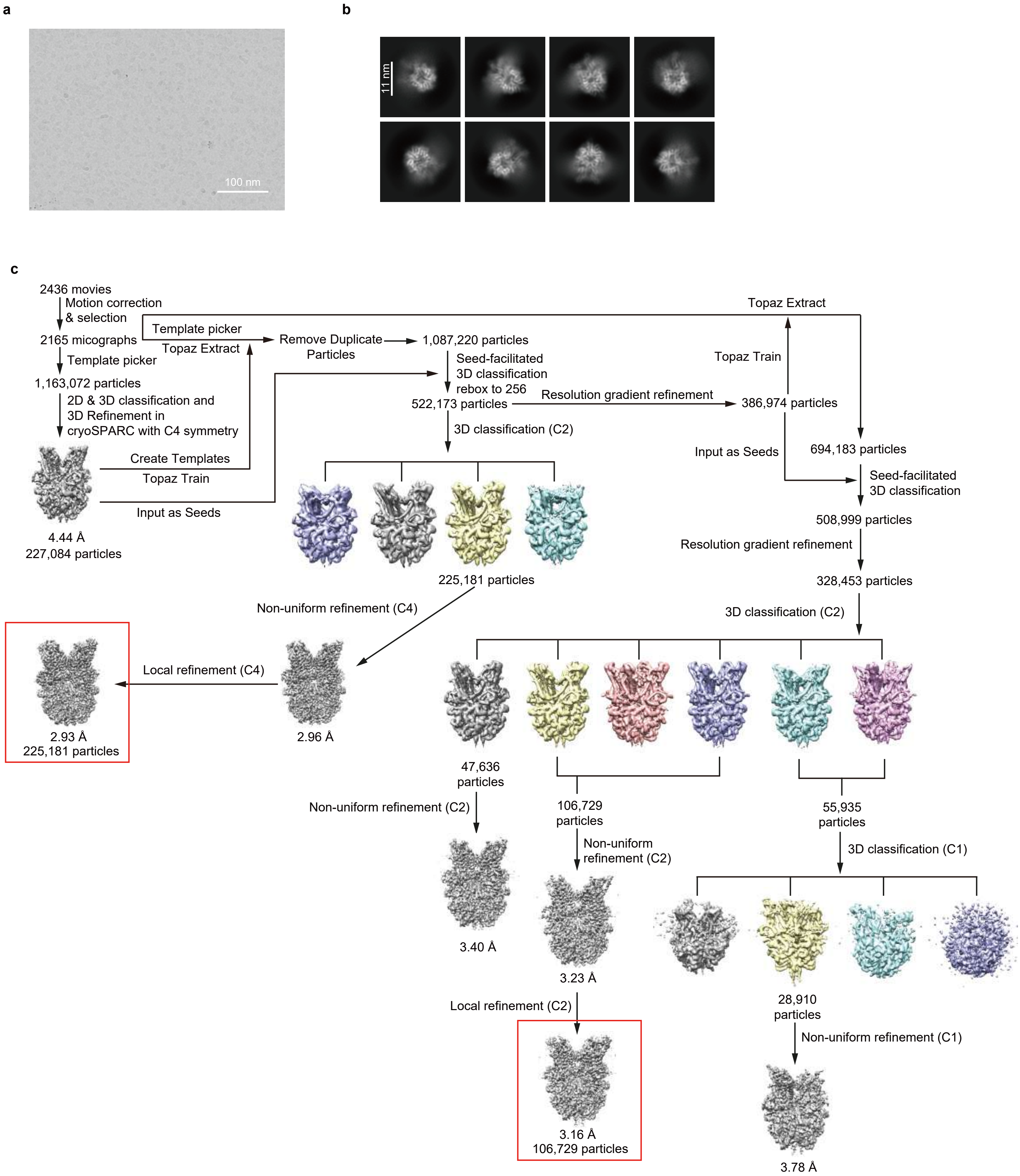

Supplementary Figure 3. Cryo-EM image analysis of the hTRPC3 T561A mutant.
a, Representative raw micrograph of hTRPC3 T561A purified using 4F-disc.
b, Representative 2D class average of hTRPC3 T561A.
c, Flowchart of hTRPC3 T561A cryo-EM data processing.

Supplementary Figure 4

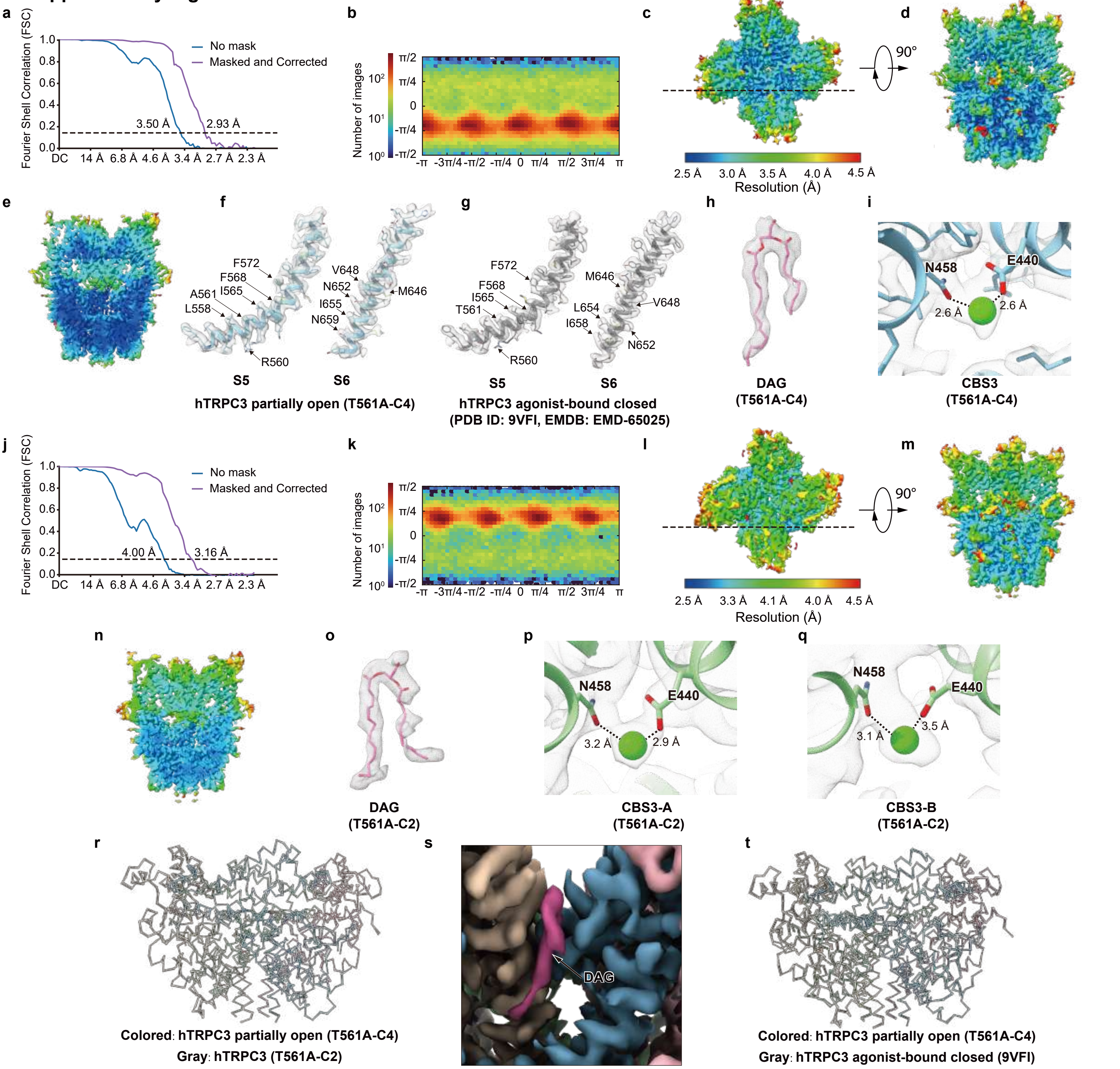

Supplementary Figure 4. Cryo-EM reconstruction of the hTRPC3 T561A mutant.

a, FSC curves of the final refinement for hTRPC3 T561A-C4 after correction for masking effects. Resolution estimations were based on the FSC 0.143 cutoff criterion.

b, Angular distribution of the final refinement for hTRPC3 T561A-C4 (standard output from cryoSPARC).

c, Local resolution estimation of hTRPC3 T561A-C4 (top view).

d, Local resolution estimation of hTRPC3 T561A-C4 (side view).

e, Cross-section of the local resolution estimation of hTRPC3 T561A-C4. The position of the cross-section is indicated by the dashed line in (c).

f, Electron density maps of hTRPC3 T561A-C4 pore helices S5 and S6 are shown in gray meshes. The contour levels were 2.4 σ.

g, Electron density maps of wild-type hTRPC3 pore helices S5 and S6 are shown in gray meshes. The contour levels were 3 σ.

h, The electron density map of DAG in the hTRPC3 T561A-C4 structure is shown as a gray mesh, contoured at 2.4 σ.

i, Close-up view of calcium-binding site (CBS) 3. Densities of ions and surrounding amino acids are shown in gray meshes, contoured at 2.4 σ

j, FSC curves of the final refinement for hTRPC3 T561A-C2 after correction for masking effects. Resolution estimations were based on the FSC 0.143 cutoff criterion.

k, Angular distribution of the final refinement for hTRPC3 T561A-C2 (standard output from cryoSPARC).

l, Local resolution estimation of hTRPC3 T561A-C2 (top view).

m, Local resolution estimation of hTRPC3 T561A-C2 (side view).

n, Cross-section of the local resolution estimation of hTRPC3 T561A-C2. The position of the cross-section is indicated by the dashed line in (l).

o, The electron density map of DAG in the hTRPC3 T561A-C2 structure is shown as a gray mesh, contoured at 2 σ.

p-q, Close-up view of calcium-binding site (CBS) 3. Densities of ions and surrounding amino acids are shown in gray meshes, contoured at 3 σ. CBS3-A and CBS3-B represent the CBS3 of subunits A and B in Fig. 1q, respectively.

r, Structural comparison of the CTD between hTRPC3 T561A-C4 and hTRPC3 T561A-C2 (gray).

s, Close-up view of the L2 DAG-binding site (boxed in Fig. 1e).

t, Structural comparison of the CTD between hTRPC3 T561A-C4 and wild-type hTRPC3 (gray, PDB ID: 9VFI).

Supplementary Figure 5

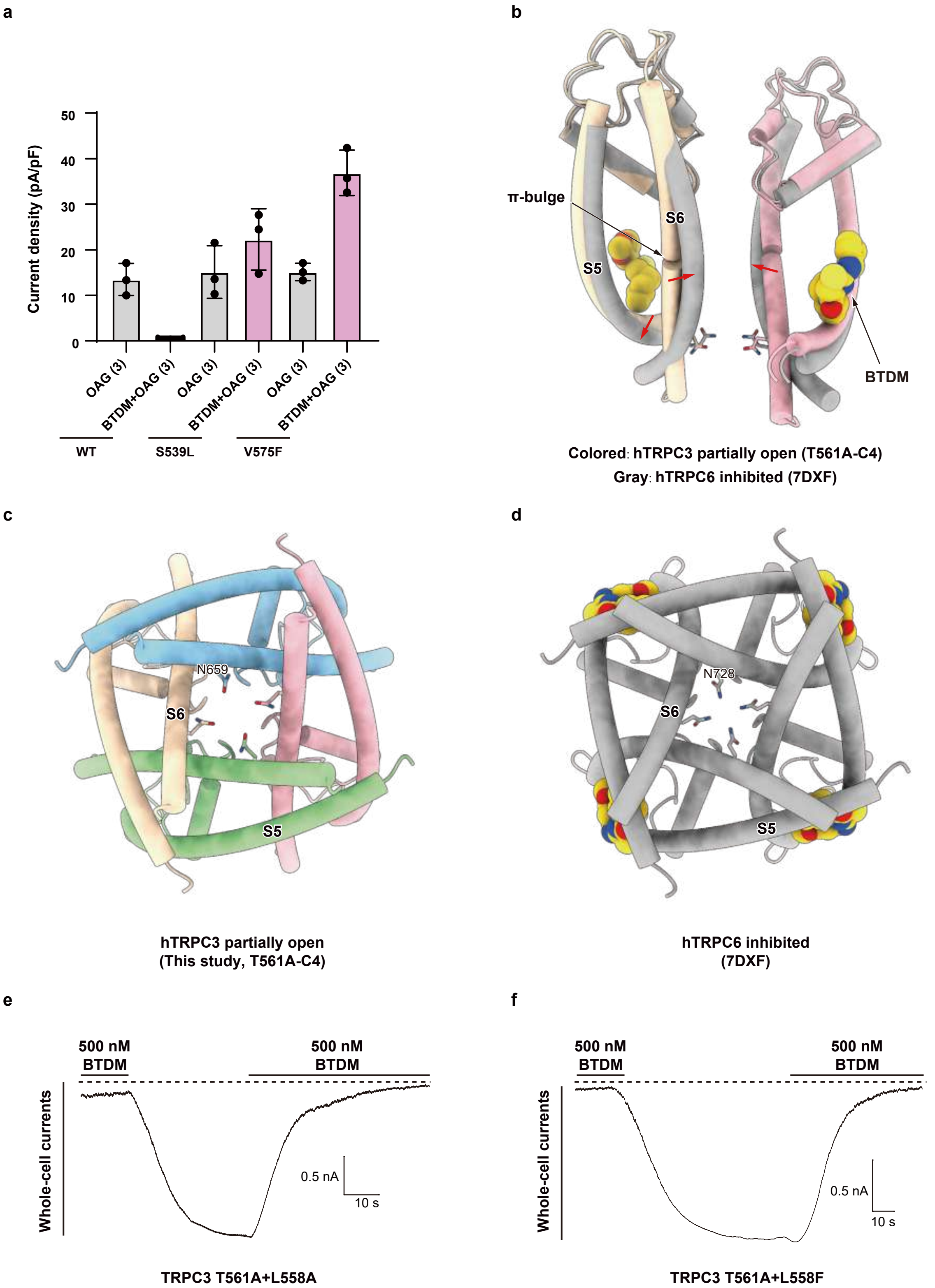

Supplementary Figure 5. Conformational changes of hTRPC3 from the partially open to the BTDM-inhibited state.
a, Whole-cell currents of hTRPC3 wild-type and hTRPC3 mutants recorded at -60 mV and 24 hours post-transfection. The concentration of OAG is 5 μM and the concentration of BTDM is 0.5 μM. Data are presented as mean ± SD. The number of replicates in each group is indicated in parentheses.
b, Structural comparison between hTRPC3 in the partially open state (T561A-C4, colored) and hTRPC6 in the BTDM-inhibited closed state (gray, PDB ID: 7DXF). Arrows denote the movement of S6. The π-bulge near the middle of S6 is marked. BTDM is shown as spheres.
c, The gate of hTRPC3 in the partially open state.
d, The closed gate of hTRPC6 in the BTDM-inhibited state.
e, Whole-cell currents of hTRPC3 T561A/L558A double mutant recorded at -20 mV and 16 hours post-transfection. The dashed line denotes the zero current level. Recordings were repeated three times with similar results.
f, Whole-cell currents of hTRPC3 T561A/L558F double mutant recorded at -20 mV and 16 hours post-transfection. The dashed line denotes the zero current level. Recordings were repeated three times with similar results.

Supplementary Figure 6

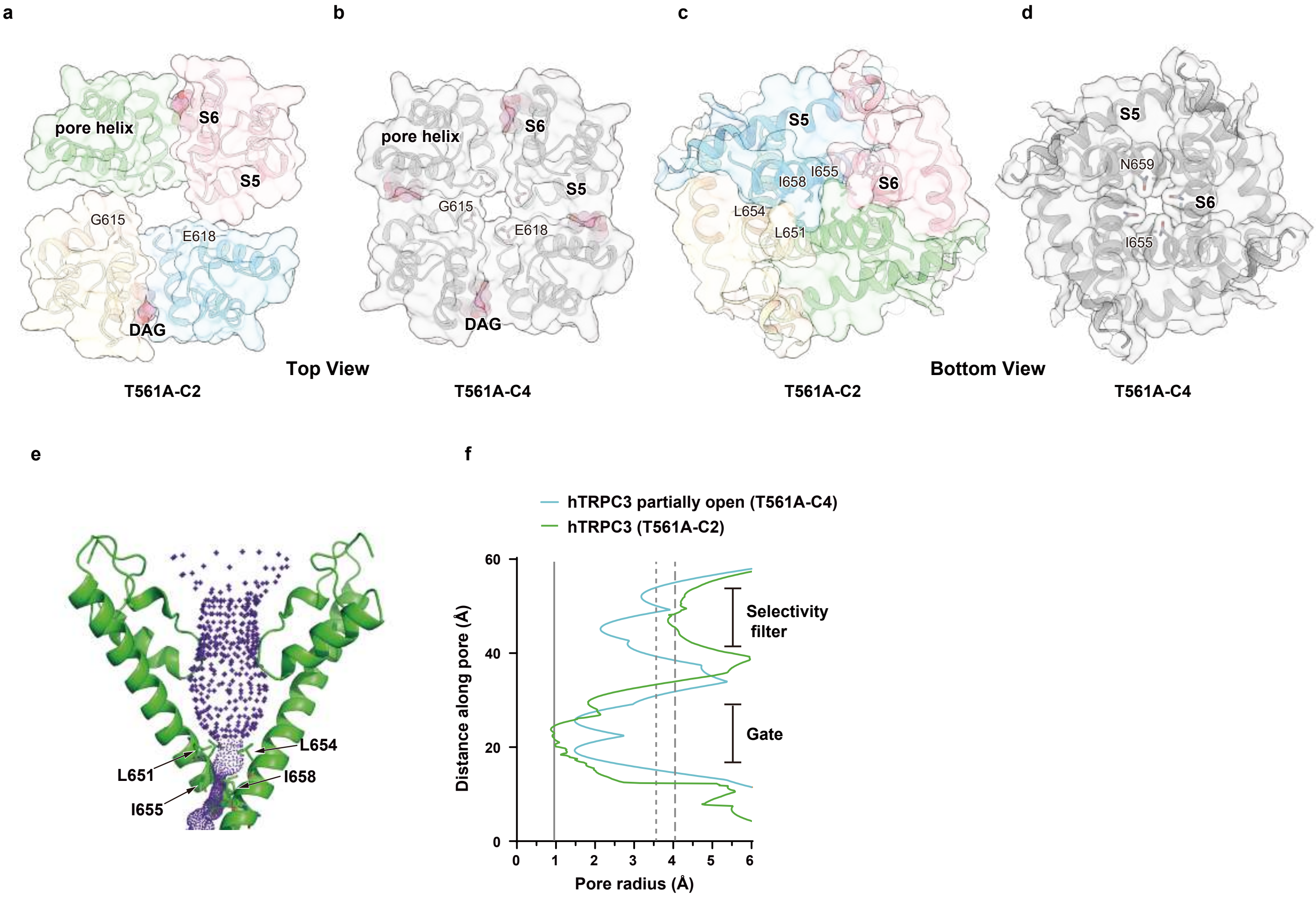

Supplementary Figure 6. DAG binding stabilizes the open state.
a, Top view of the extracellular surface of the T561A-C2 structure. DAG is shown as spheres.
b, Top view of the extracellular surface of the T561A-C4 structure. DAG is shown as spheres.
c, Bottom view of the closed gate of the T561A-C2 structure.
d, Bottom view of the partially open gate of the T561A-C4 structure.
e, Pore profile of the hTRPC3 T561-C2 structure calculated using HOLE2. Only the S6 helices of two diagonal subunits are shown for clarity. Residues at the pore constriction are shown as sticks.
f, Pore radius calculated using HOLE2. The gray solid line denotes the radius of a dehydrated calcium ion (1.0 Å). The gray dashed lines denote the radii of hydrated Na^{+} (dotted) and Ca^{2+} (dashed) ions.

Supplementary Figure 7

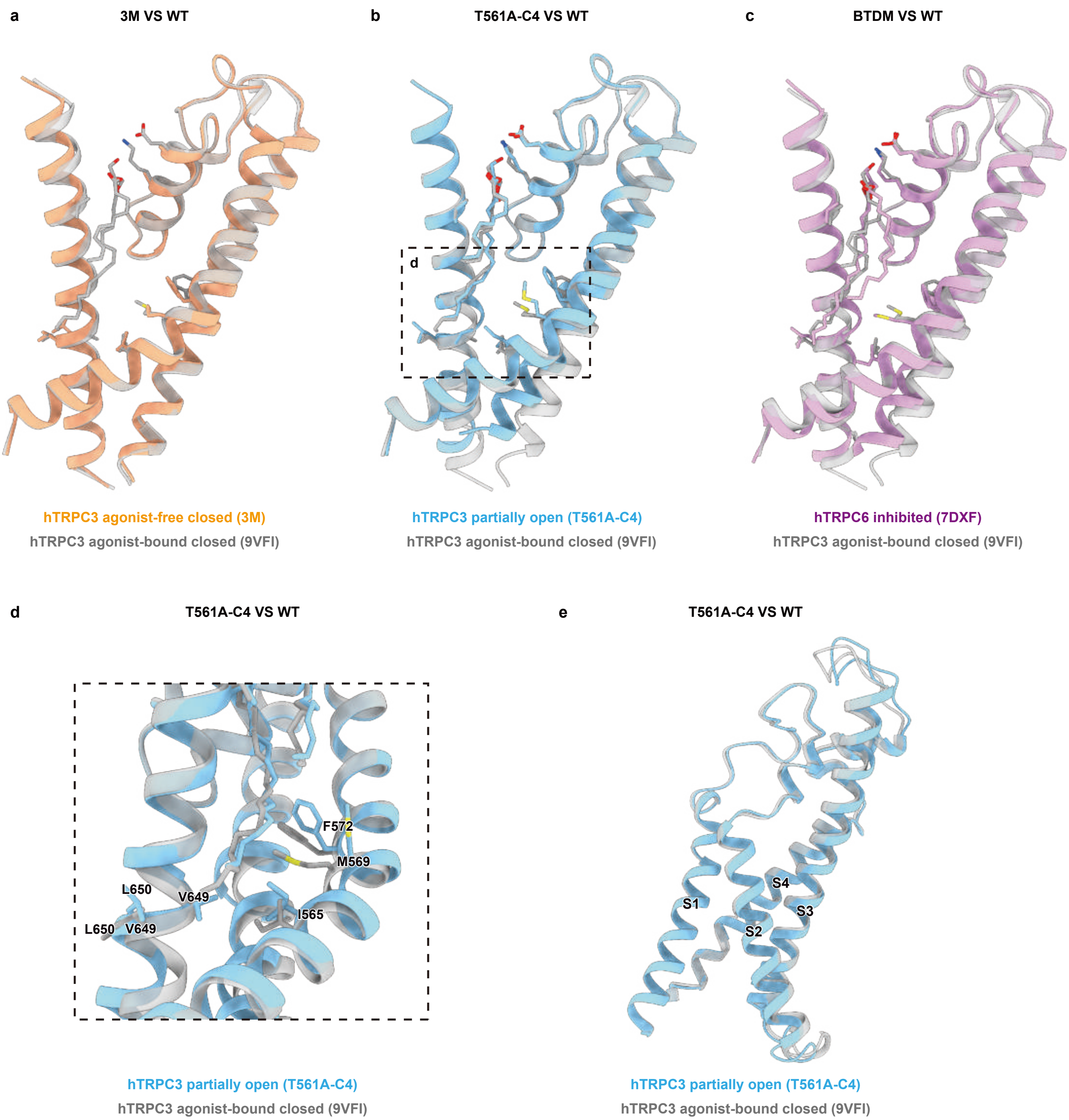

Supplementary Figure 7. Structural comparisons.

a, Structural comparison between agonist-free closed hTRPC3 (hTRPC3-3M, orange) and agonist-bound closed hTRPC3 (wild-type hTRPC3, PDB ID: 9VFI, gray) at the DAG-binding site. DAG is shown as sticks.

b, Structural comparison between partially open hTRPC3 (hTRPC3 T561A-C4, blue) and agonist-bound closed hTRPC3 (wild-type hTRPC3, PDB ID: 9VFI, gray) at the DAG-binding site.

c, Structural comparison between BTDM-inhibited hTRPC6 (PDB ID: 7DXF, purple) and agonist-bound closed hTRPC3 (wild-type hTRPC3, PDB ID: 9VFI, gray) at the DAG-binding site.

d, Close-up view of the DAG binding site of panel b near its tail. Conformational changes of side chains during channel opening are denoted.

e, Structural comparison between partially open hTRPC3 (hTRPC3 T561A-C4, blue) and agonist-bound closed hTRPC3 (wild-type hTRPC3, PDB ID: 9VFI, gray) at VSLD.

Supplementary Figure 8

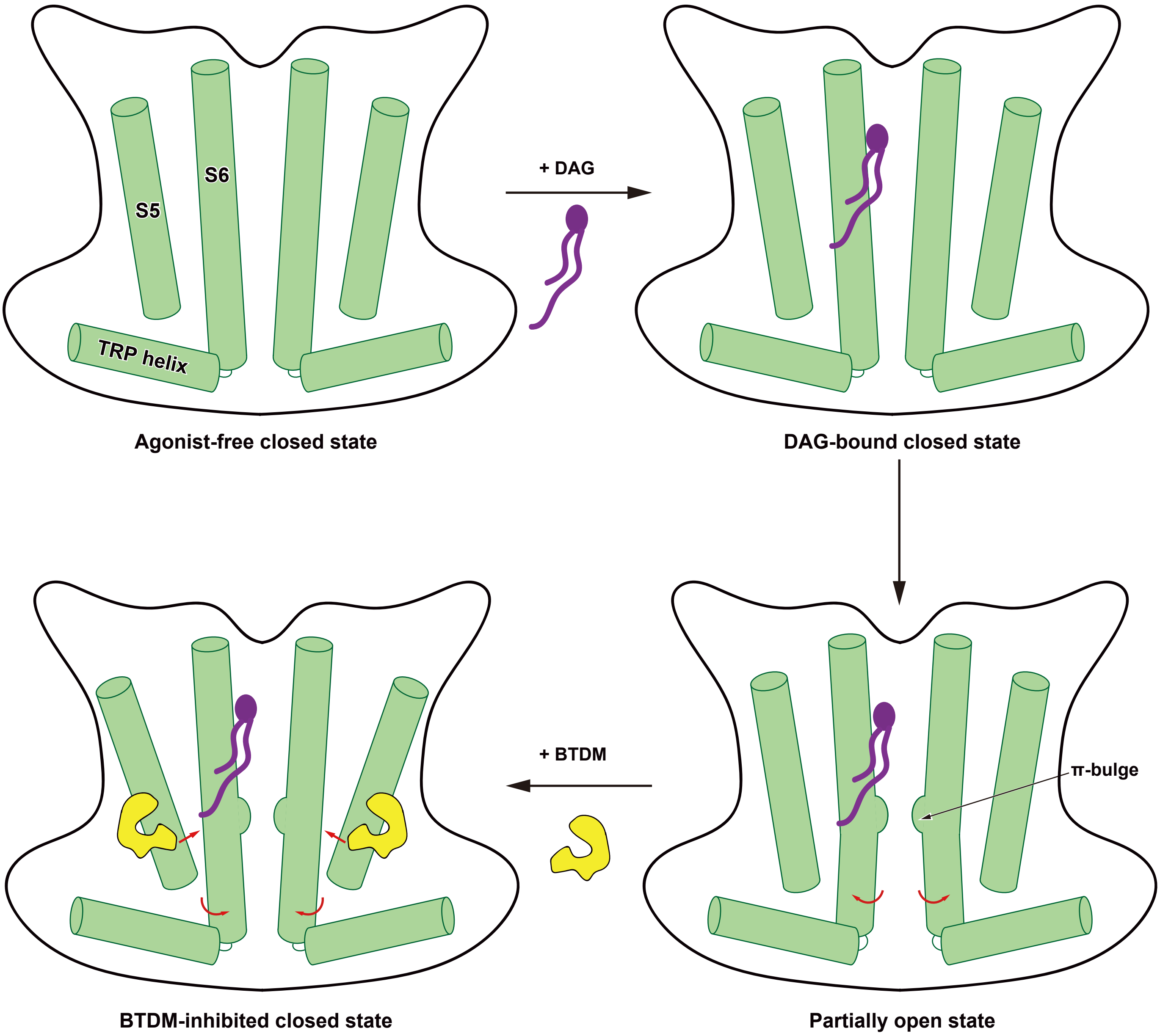

Supplementary Figure 8. The hypothetical gating model of hTRPC3 during the *in vitro* whole cell recordings.
A hypothetical cartoon model for hTRPC3 during the *in vitro* whole-cell recordings is shown. hTRPC3 transitions from the agonist-free closed state to the DAG-bound closed state, then to the partially open state, and finally to the BTDM-inhibited state. The S5 and S6 helices are shown as cylinders.

Supplementary Table 1
Cryo-EM data collection, refinement and validation statistics

	hTRPC3-3M	T561A-C4	T561A-C2
PDB ID	25CG	25CH	25CI
EMDB ID	EMD-80010	EMD-80011	EMD-80012
**Data collection and processing**			
Magnification	60,000 ×	47,000 ×	47,000 ×
Voltage (kV)	300	300	300
Electron exposure ($e^-/Å^2$)	50	50	50
Defocus range (μm)	-1.5 to -1.8	-1.5 to -1.8	-1.5 to -1.8
Pixel size (Å)	0.833	1.067	1.067
Symmetry imposed	*C4*	*C4*	*C2*
Initial particle images (no.)	160,734	1,163,072	1,163,072
Final particle images (no.)	56,124	225,181	106,729
Map resolution (Å)	2.78	2.93	3.16
FSC threshold	0.143	0.143	0.143
Map resolution range (Å)	333-2.78	273-2.93	273-3.16
**Refinement**			
Initial model used (PDB code)	7DXB	7DXB	7DXB
Model resolution (Å)	3.0	3.1	3.5
FSC threshold	0.5	0.5	0.5
Model resolution range (Å)	333-3.0	273-3.1	273-3.5
Map sharpening *B* factor ($Å^2$)	-98.6	-133.6	-122.0
Model composition			
Non-hydrogen atoms	23,000	23,312	23,168
Protein residues	2,876	2,896	3,136
Ligands	16	20	18
B factors ($Å^2$)			
Protein	37.58	72.28	106.38
Ligand	55.74	71.27	127.28
R.m.s. deviations			
Bond lengths (Å)	0.005	0.004	0.006
Bond angles (°)	0.929	0.923	0.982
Validation			
MolProbity score	1.46	1.47	1.66
Clashscore	8.61	8.77	7.97
Poor rotamers (%)	0.00	0.64	0.72
Ramachandran plot			
Favored (%)	98.73	98.60	96.53
Allowed (%)	1.27	1.40	3.47
Disallowed (%)	0.00	0.00	0.00
